# Gene expression patterns in the hippocampus during the development and aging of *Glud1* (Glutamate Dehydrogenase 1) transgenic and wild type mice

**DOI:** 10.1186/1471-2202-15-37

**Published:** 2014-03-04

**Authors:** Xinkun Wang, Nilam D Patel, Dongwei Hui, Ranu Pal, Mohamed M Hafez, Mohamed M Sayed-Ahmed, Abdulaziz A Al-Yahya, Elias K Michaelis

**Affiliations:** 1 Higuchi Biosciences Center, University of Kansas, 2099 Constant Ave., Lawrence, KS 66047, USA; 2 Alzheimer’s Disease Center, University of Kansas Medical Center, Kansas City, KS 66160, USA; 3 Department of Pharmacology and Toxicology, University of Kansas, Lawrence, KS, USA; 4 Department of Pharmacology and Toxicology, School of Pharmacy, King Saud University, Riyadh, Saudi Arabia; 5 Ibn Sina National College, Jeddah, Saudi Arabia

**Keywords:** Brain aging, Hippocampus, Glutamate, Gene expression profile, Genome

## Abstract

**Background:**

Extraneuronal levels of the neurotransmitter glutamate in brain rise during aging. This is thought to lead to synaptic dysfunction and neuronal injury or death. To study the effects of glutamate hyperactivity in brain, we created transgenic (Tg) mice in which the gene for glutamate dehydrogenase (*Glud1*) is over-expressed in neurons and in which such overexpression leads to excess synaptic release of glutamate. In this study, we analyzed whole genome expression in the hippocampus, a region important for learning and memory, of 10 day to 20 month old *Glud1* and wild type (wt) mice.

**Results:**

During development, maturation and aging, both Tg and wt exhibited decreases in the expression of genes related to neurogenesis, neuronal migration, growth, and process elongation, and increases in genes related to neuro-inflammation, voltage-gated channel activity, and regulation of synaptic transmission. Categories of genes that were differentially expressed in Tg *vs.* wt during development were: synaptic function, cytoskeleton, protein ubiquitination, and mitochondria; and, those differentially expressed during aging were: synaptic function, vesicle transport, calcium signaling, protein kinase activity, cytoskeleton, neuron projection, mitochondria, and protein ubiquitination. Overall, the effects of *Glud1* overexpression on the hippocampus transcriptome were greater in the mature and aged than the young.

**Conclusions:**

Glutamate hyperactivity caused gene expression changes in the hippocampus at all ages. Some of these changes may result in premature brain aging. The identification of these genomic expression differences is important in understanding the effects of glutamate dysregulation on neuronal function during aging or in neurodegenerative diseases.

## Background

Glutamate (Glu) is the major excitatory neurotransmitter in the mammalian central nervous system (CNS). Glutamate activation of post-synaptic receptors leads to rapid excitation of neurons as well as long-lasting effects on neuronal structure and function [1,2]. Among the long-lasting effects of Glu on neurons are changes in synaptic excitability [1,3,4], altered metabolic states and generation of reactive oxygen species in neurons [2,5-7], increases in intracellular calcium (Ca^2+^) concentrations, and upon exposure to high Glu levels, neuronal injury or cell death [8-11].

There are two other important neurobiological processes that are influenced by Glu release and the activation of neuronal Glu receptors. These are the cellular events occurring during CNS development and the neuronal changes related to the aging process. Glutamate receptor activation and the influx of Ca^2+^ are crucial to neurogenesis and the survival of neurons during early development [5,12,13], as well as to neuronal migration [14] and synaptic formation in the developing brain [15,16]. With regard to the aging process, neuronal, dendritic, and synaptic losses in hippocampus, subiculum, dentate gyrus, and pre-frontal cortex are subtle [17-19] and may be partially the result of a gradual rise in extracellular Glu in the aging brain [20-22]. An age-associated increase in the sensitivity of certain neurons to the cytotoxic effects of Glu [23] and a significant decrease in the dendrite levels of the microtubule-associated protein 2 (MAP2), a marker protein of dendrite structure, have been described [24,25]. Given the strong relationship between Glu hyperactivity and decreases in MAP2 labeling in dendrites of sensitive neurons [26-28], the MAP2 decreases in aging brain may represent a sign of increased Glu activation of receptors in susceptible neurons.

The study of neuronal responses to persistently high levels of Glu activity at synapses during development and aging requires the use of transgenic or null mutant animals that either exhibit diminished Glu re-uptake into neurons and glial cells [29-31] or have increased synaptic Glu release [32]. Null mutant mice for the high affinity glial Glu transporter genes *Slc1a2* (*Solute carrier family 1 member 2*) and *Slc1a3* exhibit high levels of extracellular Glu and suffer extensive brain damage and embryonic lethality [29-31]. Null mutants for the gene *Tsc1* (*Tuberous sclerosis complex-1*), a gene that is closely associated with the expression and function of Glu transporters in the CNS, also have high extracellular levels of Glu and suffer from extensive neuronal damage, intractable seizures, and marked reduction in their lifespan [33]. Therefore, none of these mice would be suitable for studies of both developmental and aging effects of excess Glu synaptic activity on brain cells. On the other hand, transgenic (Tg) mice over-expressing the gene for Glu dehydrogenase 1 (*Glud1*), a mitochondrial matrix enzyme, only in CNS neurons have lifelong excess synaptic release of Glu, selective neuronal degeneration in vulnerable brain regions, such as the hippocampus, and a close to normal lifespan [32,34].

The *Glud1* Tg mouse was selected to further characterize the effects of moderate excess Glu activity on gene expression patterns in the developing, adult, middle-aged, and aging mouse brain. Whole genome transcription patterns represent an unbiased assessment of changes in genes involved in brain metabolism, neuronal excitability, neuronal structure, and the response to metabolic, oxidative and other types of stresses related to Glu hyperactivity throughout development, adulthood and aging. In the present study, we determined the gene expression patterns in Tg and wt mice from 10 days to 20 months of age.

## Results and discussion

We performed genome wide transcriptomic analyses of the hippocampus at 5 ages, from early development at 10 days postnatal, to young adult (4.5 months), adult (9 months), middle aged (14.5 months), and old age (20 months). We included a group of very young mice, the 10 day old mice, so that we may assess the effects of the over-expressed *Glud1* gene on the overall patterns of gene expression during the development of Tg and wt mice. The brain region of interest was the hippocampus.

### Transcriptomic similarities between Glud1 Tg and wt mouse hippocampus from 10 days to 20 months of age

**Figure 1:**
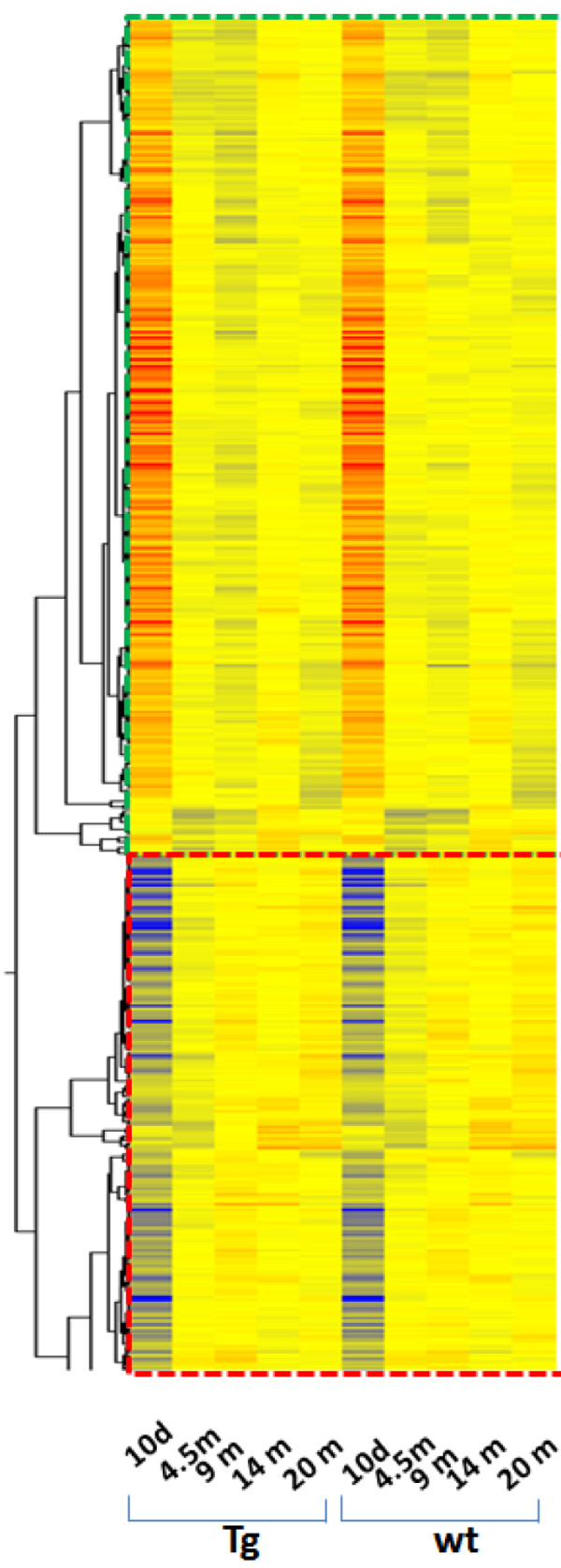
**Hierarchical clusters of genes that were expressed in *Glud1 *Tg and wt hippocampus with nearly identical patterns of change during development and aging.** Upper panel (demarcated by red dashed line) includes the genes whose expression decreased with age, while the lower panel shows genes whose expression increased with age.

Of the 45,037 gene probesets on the Affymetrix GeneChip arrays (Mouse Genome 430 2.0) that we employed in this study, we identified 895 genes whose expression was changing during development, maturation, and aging, and whose patterns of age-related changes were similar in both Tg and wt hippocampus. In this group of 895 genes, the majority were genes whose expression levels decreased with advancing age. The expression of only a relatively small fraction of the 895 genes increased gradually with age.As shown in Figure 1, the genes that exhibited similar age-related changes in wt and Tg mice can be separated into two clusters. The two clusters were characterized as consisting of: 1) genes with high levels of expression in the 10-day old hippocampus but whose expression decreased with advancing age; and 2) of genes with low levels of expression in the 10-day old hippocampus but whose expression increased with advancing age. To determine which functions are associated with the genes whose expression diverged, the Gene Ontology (GO) categories significantly enriched with such genes were identified.

For the genes whose expression in hippocampus decreased with advancing age, the biological functions in the GO categories were: 1) *Cytoskeleton* (including actin filament and microtubule); 2) *Neurogenesis*; 3) *Neuron projection/growth cone/axon guidance/axonogenesis*; 4) *Small-GTPase-mediated signal transduction; 5) Cholesterol biosynthesis*; 6) *Cell migration*; 7) *Cell cycle and cell division*; and 8) *Protein disulfide oxidoreductase activity and cell redox homeostasis*. For the genes whose expression increased with advancing age, the significantly enriched GO categories included the following: 1) *Axon and neuron projection*; 2) *Actin binding*; 3) *Small GTPase regulator activity*; 4) *Voltage-gated channel activity* (mostly potassium); 5) *Protein tyrosine kinase activity*; and 6) *Regulation of synaptic transmission*. It was noted that two function categories, *Cytoskeleton structure and function* and *Axon and neuron projection*, contained genes that exhibited an age-associated up-regulation as well as genes whose expression was down-regulated with age. In addition to the biological functions mentioned above, a specific biological pathway was identified as being enriched with genes whose expression increased with advancing age, the *Chemokine signaling* pathway.

The overall patterns of gene changes with age in both wt and Tg mouse hippocampus could be viewed as being characterized by a decline in aspects of neurogenesis, neuronal migration, cytoskeleton function, cholesterol biosynthesis, and cell redox regulation. While on the other hand, neuroinflammation, protein tyrosine kinase signaling, and voltage-gated potassium channel activity were functions that increased during the transition from newborn to adult and to old age. Increases with advancing age in the expression of genes related to neuro-inflammation are consistent with previous observations of increases in the process of inflammatory response during brain aging and in neurodegenerative diseases [35].

### Transcriptomic differences between Glud1 Tg and wt mouse hippocampi across the ages from 10 days to 20 months

The next issue that we explored was whether there were differences in age-associated gene expression in the hippocampus of wt *vs.* Tg mice. We identified 407 genes that were differentially expressed in Tg *vs.* wt mouse hippocampus. We noted that the *Glud1* gene was not among the differentially expressed genes. The reason for that is related to the fact that the two sets of probes for *Glud1* on the GeneChip are designed to hybridize with the 3’-UTR region of the gene transcript, a region that was not present in the transgene construct that we had inserted into the mouse genome of the *Glud1* Tg mice. Regardless of the lack of detection of excess *Glud1* expression using the microarray chips, we have documented in our previous studies the insertion of the *Glud1* open reading frame in the genomic DNA of the transgenic mice, as well as the over-expression of the GLUD1 protein and increased glutamate dehydrogenase activity in neurons [32], thus confirming the presence and expression of the *Glud1* transgene.

The major functions of the differentially expressed genes are summarized in Table 1. Among these functions, *Mitochondrial envelop* and *Carboxylic acid transport* contain genes in the nuclear genome that code for proteins related to mitochondrial metabolism and function. These genes included: *Crls1*, coding for an enzyme that catalyzes the biosynthesis of di-phosphatidyl glycerol (cardiolipin) in mitochondria; *Cds1* and *Cds2*, coding for enzymes that catalyze the conversion of phosphatidic acid to CDP-diacylglycerol, a precursor to the synthesis of phosphatidyglycerol and cardiolipin; and *Cox4i2, Cox15,* and *Cox5A*, genes coding structural and regulatory components of the multimeric complex that forms the mitochondrial enzyme cytochrome c oxidase (Table 1). Cytochrome c oxidase is a key enzyme in the electron transport system of mitochondria and one that has been reported to be decreased in terms of expression levels and activity during aging and in Alzheimer’s disease [36,37]. The differential expression of these mitochondria-related genes during development, maturation, and aging were suggestive of distinctive patterns of activity between wt and Tg mouse hippocampus cells in two key mitochondrial metabolic pathways, lipid biosynthesis and oxidative phosphorylation.

**Table 1:** **Bio-functions (gene ontology terms) of differentially expressed genes in *Glud1 *** **Tg *vs. *** **wt hippocampus across 5 ages**

**Term**	**P value**	**Key genes**
Ras protein signal transduction	0.0016	Bcl6, Brap, Eps8l1, Hrasls, Nras, Ptplad1, Sdcbp
Metal ion transport (*mostly for calcium and potassium*)	0.0017	Atp1a2, Atp1b1, Atp2c1, Cacna1h, Cacnb4, Camk2g, Itpr2, Kcnab2, Kcnip2, Kcnip3, Kcnk1,,Kcnq2, Kcnt1, Sfxn1, Slc25a28, Slc38a9, Trpc4
Synaptic transmission	0.013	Atp1a2, Cacnb4, Capza2, Cplx3, Grid2, Grm4, Lin7c, Stx1b
Carboxylic acid transport	0.017	Cacnb4, Serinc1, Slc13a3, Slc1a1, Slc1a7, Slc38a9, Trpc4,
Actin cytoskeleton organization	0.027	Acta1, Arhgef2, Atp2c1, Bcl6, Capza2, Dbn1, Neurl2, Nras, Sorbs1
Stress-activated protein kinase signaling pathway (*mostly MAPK and JNK cascades*)	0.034	Brap, Cdc42se1, Errfi1, Fgf12, Fgf2, Gm8188, Map3k7, Myd88, Ptplad1
Mitochondrial envelop	0.05	Cds1, Cds2, Cox15, Cox4i2, Cox5a, Crls1, Cyb5b, Golph3, Nlrx1, Pi4kb, Ppp1cc, Sfxn1, Slc25a28, Tomm20

In addition to the genes that code for mitochondrial proteins, another group of important genes shown in Table 1 are those of *Metal ion transport*, in particular, those for potassium channels. Differential expression of these genes would lead to differential regulation of neuronal excitability, sensitivity to Glu-induced neuronal damage, and neurocognitive function. Two of these genes were *Kcnq2* (also known as the M channel) and *Kcnt1*, a sodium and calcium-regulated potassium channel. Mutations in these two genes are associated with early onset epileptic seizures [38,39]. A third potassium channel gene was *Kcnab2*, a gene that codes for the beta subunit of voltage-gated potassium channels and whose over-expression protects neurons from Glu-induced cell damage [40] while its under-expression impedes associative memory formation [41].

Of all differentially expressed genes, we identified a subgroup of eight genes which exhibited the most significant differences. These eight genes were: *Akt3*, *Cebpg*, *Klhdc8a*, *Pex11b*, *Prrt4*, *Sfxn1*, *Tomm20*, and *Ubr7. Akt3*, a serine/threonine protein kinase (protein kinase B), is an important component of many signal transduction pathways. *Akt3* is involved in the regulation of cell proliferation, tumorigenesis, differentiation, organismal development, metabolism, synaptic transmission, and cell junction formation [42]. *Cebpg* (CCAAT/enhancer binding protein gamma), a transcription factor, is associated with stress pathways including antioxidant, DNA repair, and immune responses [43,44]. *Klhdc8a*, one of the Kelch domain-containing proteins that are connected to the cytoskeleton, is related to neurite outgrowth and to the development of drug-resistant forms of gliomas [45,46]. *Pex11b* (peroxin 11b), a peroxisomal biogenesis factor, interacts with proteins involved in mitochondrial fission, is involved in lipid metabolism, myelin formation and axonal growth, and mutations in this and related *Pex* genes can lead to the inherited neurological and behavioral syndrome known as the Zellweger syndrome [47,48]. *Prrt4* (proline-rich transmembrane protein 4), a gene related to *Prrt2*, may be associated with abnormal neurological conditions as mutations in *Prrt2* lead to paroxysmal neurological states characterized by seizures and dyskinesias [49]. *Sfxn1* (sideroflexin 1) is a mitochondrial inner membrane tricarboxylate and iron carrier [50]. *Tomm20* (translocase of outer mitochondrial membrane 20 homolog), a component of a receptor-translocase complex in the outer mitochondrial membrane, is involved in recognition and subsequent transport into mitochondria of precursor proteins synthesized in the cytoplasm [51-53]. The differential expression across age of the two mitochondrial genes *Sfxn1* and *Tomm20* in *Glud1 vs.* wt mouse hippocampi might be further evidence of functional differences between wt and Tg mouse brain mitochondria. Finally, the differential expression patterns of *Ubr7* (ubiquitin protein ligase E3), a member of the family of *N*-terminal ubiquitin ligases that are involved in the regulation of cellular and organismal processes such as apoptosis, neurogenesis, and learning and memory [54-56], might be an indication of differential patterns of folding and degradation of select proteins in Tg *vs.* wt mice.

### Age-related periods with the highest and lowest differential gene expression between Tg and wt mouse hippocampi

**Figure 2:**
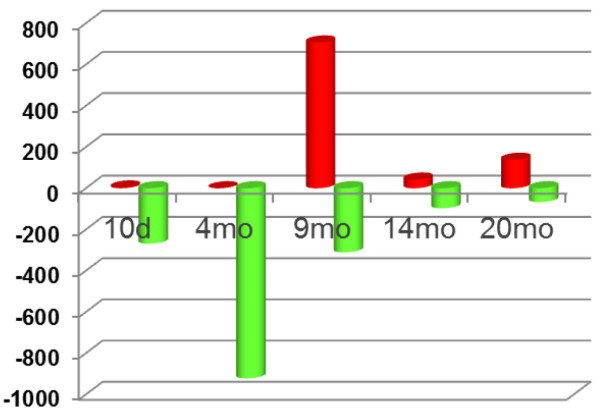
**Total number of genes (probesets) in *Glud1 *** **Tg hippocampi that were either significantly under (green)- or over (red) -expressed when compared with wt hippocampi at each age examined.**

Among the 407 differentially expressed genes, the differences between the Tg and wt mouse hippocampi were not always in one direction, *i.e.*, either over-expression or under-expression of the genes in one mouse genotype *vs.* the other. An analysis of the total number of genes whose expression in hippocampus differed in Tg *vs.* wt mice across the five ages is shown in Figure 2. In the hippocampus of 10 day-old mice, there were relatively few differences in gene expression between *Glud1* and wt mice, and most of the genes that were differentially expressed were at lower levels (down-regulation) in the Tg compared with the wt hippocampus. By 4.5 months of age, the number of genes whose levels of expression were lower in Tg than wt hippocampus had quadrupled in comparison with the 10 day old mice but, once again, only few genes were expressed at higher levels (up-regulation) in Tg than wt mice at that age (Figure 2). This pattern reversed dramatically at 9 months of age, with a high number of genes being up-regulated in Tg compared with wt hippocampus. Finally, in the last two age groups— *i.e.* the 14.5 and 20 month old mice, the populations of differentially expressed genes were relatively small, approximately at the same levels as those at 10 days of age (Figure 2).

The high number of genes that were differentially transcribed in 4.5 and 9 month-old Tg *vs.* wt mouse hippocampus might suggest that this period represented a critical stage in the maturation of the mouse hippocampus and that the over-expression of *Glud1* in neurons may have had the greatest effect around this age period. The GO categories enriched with genes that were differentially expressed in Tg *vs.* wt mice at 4.5 and 9 months of age, revealed several key neurobiological functions that were down-regulated at 4.5 months but up-regulated at 9 months in Tg *vs.* wt mouse hippocampus (Table 2). These functions were summarized as Neuronal growth and development (including *neuron projection* and *nervous system development*), Axonal path-finding (including *axonal guidance*, *cytoskeleton*, *focal* and *cell adhesion*), and Synapse formation and signaling (including *synaptic vesicle* and *synaptic transmission*).

**Table 2:** **Bio-functions of down- or up-regulated genes in *Glud1 *** **Tg compared with wt hippocampus at various ages**

**Age group**	**Down-regulated**	**Up-regulated**
10 days post birth	Ion binding and transport, transmembrane transport, extracellular space, neuronal development, metabolism and energy generation	
4.5 months	Neuron projection, axonal guidance, synaptic transmission, ion binding, cytoskeleton, protein ubiquitination, intracellular transport, cell adhesion, synaptic vesicle	
9 months	Signal transduction, nervous system development, cell adhesion, synaptic transmission, ion homeostasis	Nervous system development, signal transduction, cytoskeleton, focal adhesion, ion transport, synaptic transmission, protein folding and degradation
14.5 months	Extracellular region, cell adhesion, and ion transport	
20 months	Voltage-gated channel	Synapse

The group of genes related to neuronal growth and development consisted of the transcription factors *Fos*, *Pbx1*, *Zeb2*, and *Egr2. Fos,* FBJ murine osteosarcoma viral oncogene homolog, is the first immediate early gene identified to have an increased level of expression following neuronal stimulation in brain and it, together with other regulatory factors and immediate early genes, may be involved in dendrite growth and synaptic plasticity [57,58]. *Pbx1*, pre-B-cell leukemia homeobox 1, is a homeodomain gene involved in early neuronal development, axon pathway finding, and regulation of compulsive behaviors [59-61]. *Zeb2*, zinc finger E-box binding homeobox 2, is a gene that is involved in early brain development and the regulation of myelination of neurons; mutations of this gene lead to microcephaly, agenesis of corpus callosum, and mental retardation [62]. *Egr2*, early growth response 2, is a gene involved in axonal growth and myelination and mutations in this gene are associated with congenital neurological diseases characterized by hypo-myelination and abnormal axonal growth and function in the peripheral nervous system [63]. Other differentially expressed genes related to neurite growth, pathway finding, and synapse formation included the gene *Ndel1* coding for a cytoskeleton-organizing protein that controls neuron migration and outgrowth [64], the gene *Nrca* involved in neuronal cell adhesion, axonal growth and directional migration [65], and the gene *Ntrk2* coding for the receptor of brain-derived neurotrophic factor (BDNF) and involved in dendritic spine growth and synapse formation [66,67]. The lower levels of expression of these genes in 4.5-month-old Tg as compared with wt mouse hippocampus might be an indication of delayed neuronal growth, axonal projection, and synapse formation in the Tg mice.

At ages past 9 months, the differences in gene expression between the *Glud1* and wt mouse hippocampi were not as pronounced as at 4.5 and 9 months. Functional analyses of the GO categories significantly enriched with differentially expressed genes at these ages were indicative of some important neurobiological functions that differed between Tg and wt mouse hippocampi, such as *cell adhesion and extracellular region*, *ion transport, voltage-gated channel, and synaptic activity* (Table 2).

### Transcriptomic changes in the hippocampus during “developmental” and “aging” stages of life

The results of analyses of gene expression patterns outlined above were obtained by treating the data as if the age-associated changes in expression in Tg and wt hippocampi were part of a biological process that is expressed in a continuum from 10 days post-natal to 20 months of old age. But, we know that the immediate post-natal period up to 2-3 months of age is characterized by many changes in tissues, especially in the brain, that would be considered as developmental events. Similarly, the changes in cell and organ function with advancing age from 14.5 to 20 months would be considered part of organismal aging. For this reason, we decided to re-analyze the data on gene expression as falling into two phases in the life spectrum, a “developmental” and an “aging” stage of life. The age of 9 months appeared to represent an approximate midpoint between these two stages. Therefore, the transcriptomic data were re-analyzed by dividing them into a “developmental stage” that encompassed the period between 10 days and 9 months of age, and an “aging stage” between 9 and 20 months.

**Figure 3:**
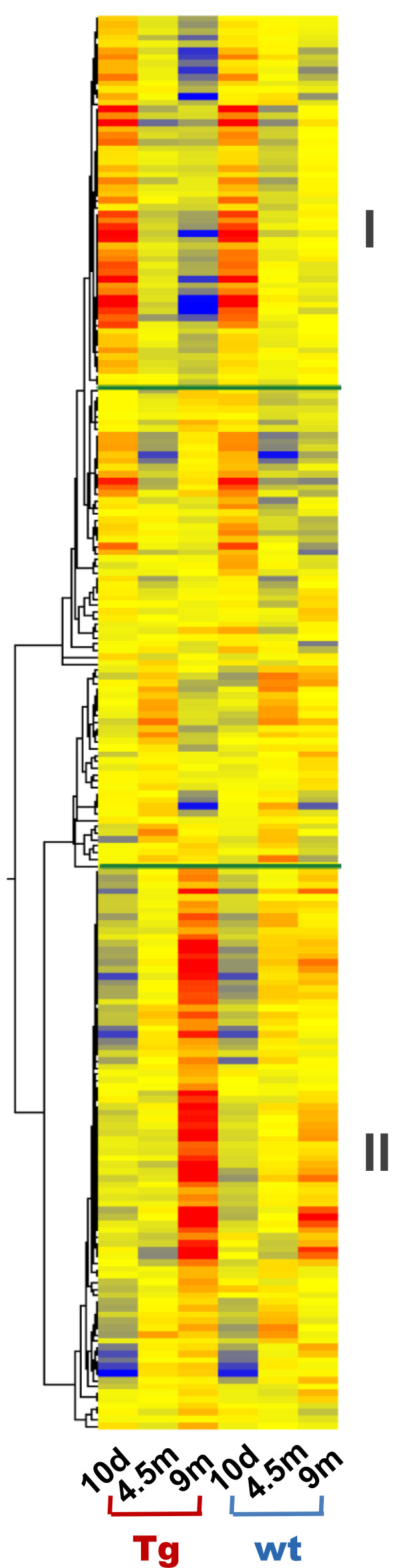
**Hierarchical clustering of genes that were differentially expressed in the Tg *vs*. wt hippocampi during the “developmental stage” (10 day, 4.5 and 9 month old).** The clusters are further subdivided into 3 gene expression patterns that were similar in Tg and wt hippocampi even though there were differences in ov: those decreasing in expression levels with advancing age and with the expression in the Tg mice at 9 months being lower than the wt (panel I); those with increasing expression with advancing age and with the Tg expression levels being higher than the wt at 9 months (panel II); and those with a mixed pattern of age-related change in expression (middle panel).

In the developmental stage, our analysis identified 217 genes which exhibited both age-dependent changes and significant expression differences between the *Glud1* and wt mice. Hierarchical clustering of these genes according to changes in their expression from 10 days to 4.5 and 9 months of age is shown in Figure 3. The top group shown in Figure 3 (group I) had high levels of expression at 10 days of age and progressively lower levels at 4.5 and 9 months. The decrease in the expression of these genes in the Tg hippocampus at 9 months was much greater than that detected in wt mice. A GO analysis of the genes in this group indicated that the biological functions significantly enriched with these genes were *RNA recognition and binding, Calcium and other metal ion binding, Protein kinase activity, Cytoskeleton,* and *Synapse*.

The bottom group of genes shown in Figure 3 (group II), had a pattern of increasing expression with advancing age from 10 days to 9 months. These genes were also differentially expressed in Tg *vs.* wt mouse hippocampus. The expression levels of these genes at 9 months of age were significantly higher in Tg compared with wt. The GO categories enriched with these genes were: *Synaptic transmission, Neuron projection and cytoskeleton, Endomembrane system* (including nuclear envelop and Golgi apparatus), *Metal ion binding and channel activity, Protein catabolic process, Cell adhesion, Plasma membrane, Mitochondrion, and Regulation of apoptosis*. The expression changes for these genes suggested that during the developmental stage, the hippocampus of the *Glud1* mice differed from that of the wt in terms of important neuronal structural and functional aspects, such as neuronal projection and neurite growth, protein degradation, mitochondrial function, and metal ion binding and transport. At the age of 9 months, the cells of the hippocampus in the Tg mice had exceeded in the expression of most of these genes the levels observed in the wt hippocampus.

**Figure 4:**
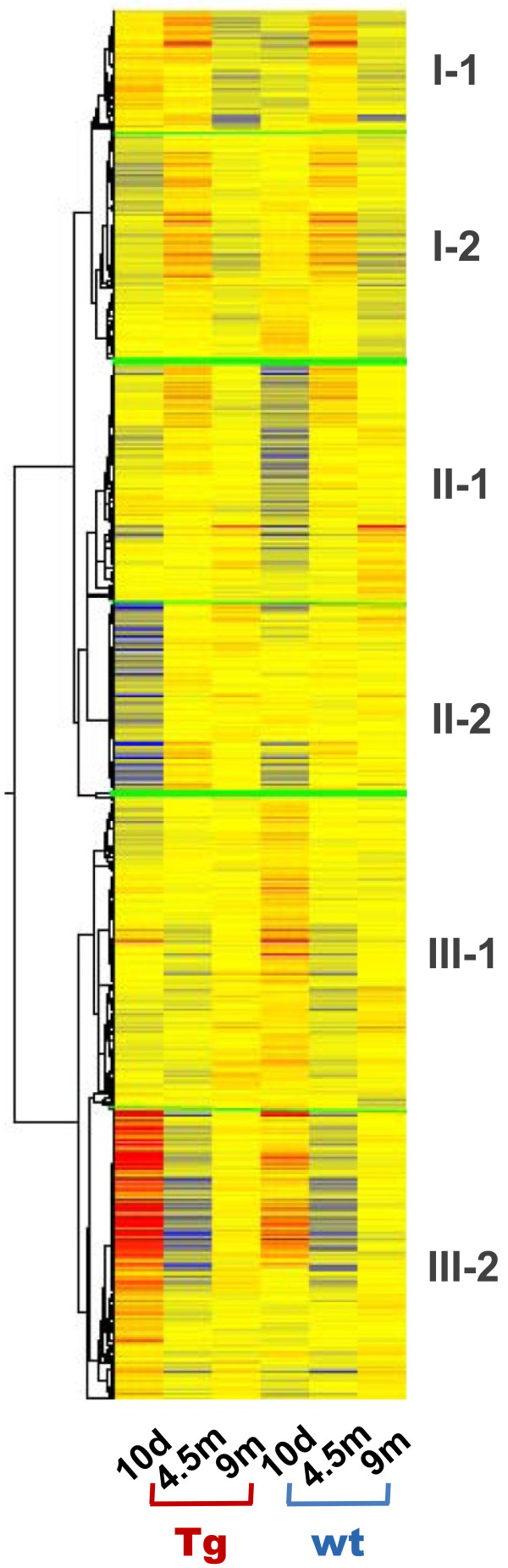
**Hierarchical clustering of genes that were differentially expressed in the Tg *vs*. wt hippocampi during the “aging stage” (9, 14.5, and 20 month old).** The clusters are further subdivided into differing gene expression patterns across age of both Tg and wt mice. A further subdivision of the clusters was made based on whether the gene expression levels for the Tg hippocampi were higher at 9 months than those for the wt (see text for full description).

A much greater number of probesets (1,257) than those that were differentially expressed in the “developmental stage” showed differential expression between *Glud1 vs.* wt hippocampus during the “aging stage”, *i.e.*, from 9 to 20 months of age (Figure 4). Only few of these genes had a continuously increasing or decreasing pattern of expression from 9 to 20 months of age. Most genes fell into one of two major groups: those whose expression increased between 9 and 14.5 months and then decreased at 20 months of age (marked as Group I in Figure 4), and those whose expression decreased between 9 and 14.5 months and then increased again at 20 months (Group III in the figure). Genes in Group I were further separated into two subgroups based on their relative expression levels in Tg *vs.* wt at the start of this aging stage (marked as Subgroups I-1 and I-2 in Figure 4). Genes in Subgroup I-1 were expressed at higher levels in Tg than wt at 9 months of age, and the GO categories significantly associated with these genes included *Cytoskeleton*, *Golgi apparatus*, *Vesicle-mediated transport*, *Calcium ion binding*, and *Protein transport*. Since three of these GO categories are related to intracellular transport processes, the expression pattern of these genes might represent possible adaptations of cells in the Tg mouse hippocampus that would lead to increased growth or elongation of processes. Genes in Subgroup I-2 were expressed at lower levels in Tg than wt at 9 months, and the GO categories associated with these genes included *Regulation of transcription*, *Metal ion binding*, and *Protein kinase activity*, *i.e.*, suggestive of changes in intracellular signaling and regulation of protein synthesis in the *Glud1* Tg mouse hippocampus.

Group III genes, *i.e.*, those whose expression levels decreased from 9 to 14.5 months and then partially rebounded at 20 months, were similarly divided into two subgroups. The genes in Subgroup III-2 (Figure 4) were expressed at higher levels in Tg than wt at 9 months of age, and the GO categories enriched with genes in this subgroup were *RNA binding*, *Protein kinase activity and ATP binding*, *GTPase regulator activity*, *Microtubule cytoskeleton*, *Actin skeleton*, and *Neuron projection*. This grouping showed, once again, that intracellular transport and neurite projection processes, *i.e.*, microtubule and actin cytoskeleton, were at higher levels in Tg than wt hippocampi at 9 months and appeared to increase again, but moderately, at 20 months. Genes in the other subgroup (III-1) were expressed at lower levels in Tg than wt at 9 months, and the categorical GO functions included *Ribosome*, *Purine metabolism*, *Mitochondrion*, *RNA binding*, *Proteolysis*, *Protein transport*, and *Calmodulin binding*. Thus, protein synthesis and metabolism, as well as mitochondrial activity, appeared to be at lower levels in Tg than wt hippocampus at 9 mos, and increased only moderately at 20 months. Among the mitochondria-related genes in this group was *Crls1,* the gene that codes for cardiolipin synthase. Cardiolipin is almost exclusively expressed in mitochondria and is present in the inner mitochondrial membrane where it enhances the activity of the electron transport system and ATP synthesis [68]. Cardiolipin is susceptible to oxidation and loss of activity with advancing age [69], therefore, the higher expression of *Crls1* in wt compared with that in Tg hippocampus may afford greater retention of normal mitochondrial function in wt than Tg mice during the aging process. The differential levels of genes related to mitochondrial function in wt *vs.* Tg hippocampus, appeared to be a recurring theme at different ages.

The gene categories described above were populated with genes that were transcribed either at high or intermediate levels at the start of the aging process. But, there was another group of the genes shown in Figure 4 whose expression was at low levels at 9 months but increased at 14.5 and 20 months in both Tg and wt mouse hippocampi (Group II). This group of genes could also be divided into two subgroups, one whose expression was consistently higher in Tg than wt hippocampus at 9 months (Subgroup II-1), and the other lower in Tg than wt (Subgroup II-2). For those genes in Subgroup II-1, the biological functions represented were those of *Protein ubiquitination and degradation*, *Cytoskeleton and cell projection*, *mRNA transport*, *Protein transport*, *Chaperone activity*, *Endoplasmic reticulum*, *Mitochondrion*, and *Regulation of apoptosis*. Therefore, in addition to cytoskeleton, transport, and neurite growth that were characteristic of genes enriched in the 9 month-old hippocampus of Tg mice, cell and ER stress were also enriched in this subgroup, specifically, protein ubiquitination, chaperone activity, ER, protein degradation, and regulation of apoptosis. Some of these biological functions will be analyzed further in a subsequent section. The genes in Subgroup II-2 were related to S*ynapse and synaptic vesicle*, *RNA binding*, *Cell surface receptor linked signal transduction*, *ER*, *Phosphatase activity*, *Regulation of transcription*, and *Protein transport*.

**Figure 5:**
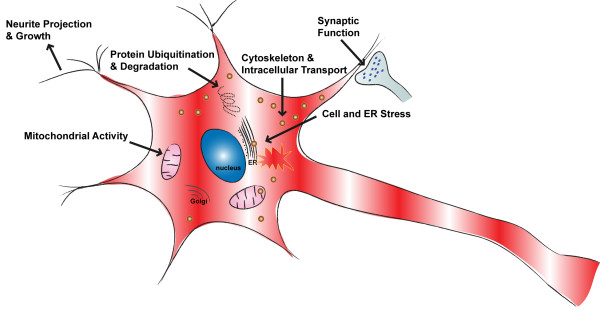
**Major functions of genes differentially regulated in *Glud1 *Tg *vs. *wt mice during the aging stage.** Most functions, including *Neurite projection and growth*, *Protein ubiquitination and degradation*, *Cytoskeleton and intracellular transport*, *Cell and ER stress*, and *Mitochondrial activity*, were up-regulated in the *Glud1* Tg mice.

As summarized in Figure 5 on the effect of *Glud1* transgene overexpression on mouse hippocampal aging, neurite projection and growth, protein ubiquitination and degradation, cytoskeleton and intracellular transport, cellular and ER stress, and mitochondrial activity were the major functional categories associated with genes whose expression levels were up-regulated in the Tg mice. Some genes related to mitochondrial activity were also down-regulated in the aging stage, as was true also with respect to genes related to synaptic function.

Combining the results from all ages, encompassing both the developmental and aging stages, allowed us to identify five categories of cell functions that were consistently and significantly differentially regulated in Tg *vs.* wt hippocampus. The first three were *Cytoskeleton, Intracellular transport*, and *Neurite projection and growth*. Aspects of differential expression of cytoskeleton and neurite projection genes in the hippocampus of Tg as compared with those of wt mice were described previously for a single age group, the 9 month old group [70] and will be analyzed further in subsequent studies focused on the function of axonal transport in the brain and hippocampus (P. Lee, R. Pal, X. Wang, I-Y. Choi, and E. K. Michaelis, unpublished observations). The other two key functions were *Protein ubiquitination and degradation,* and *Mitochondrial structure and function,* which are analyzed next.

### Differential gene expression of protein ubiquitination and degradation in Tg vs. wt hippocampus

**Figure 6:**
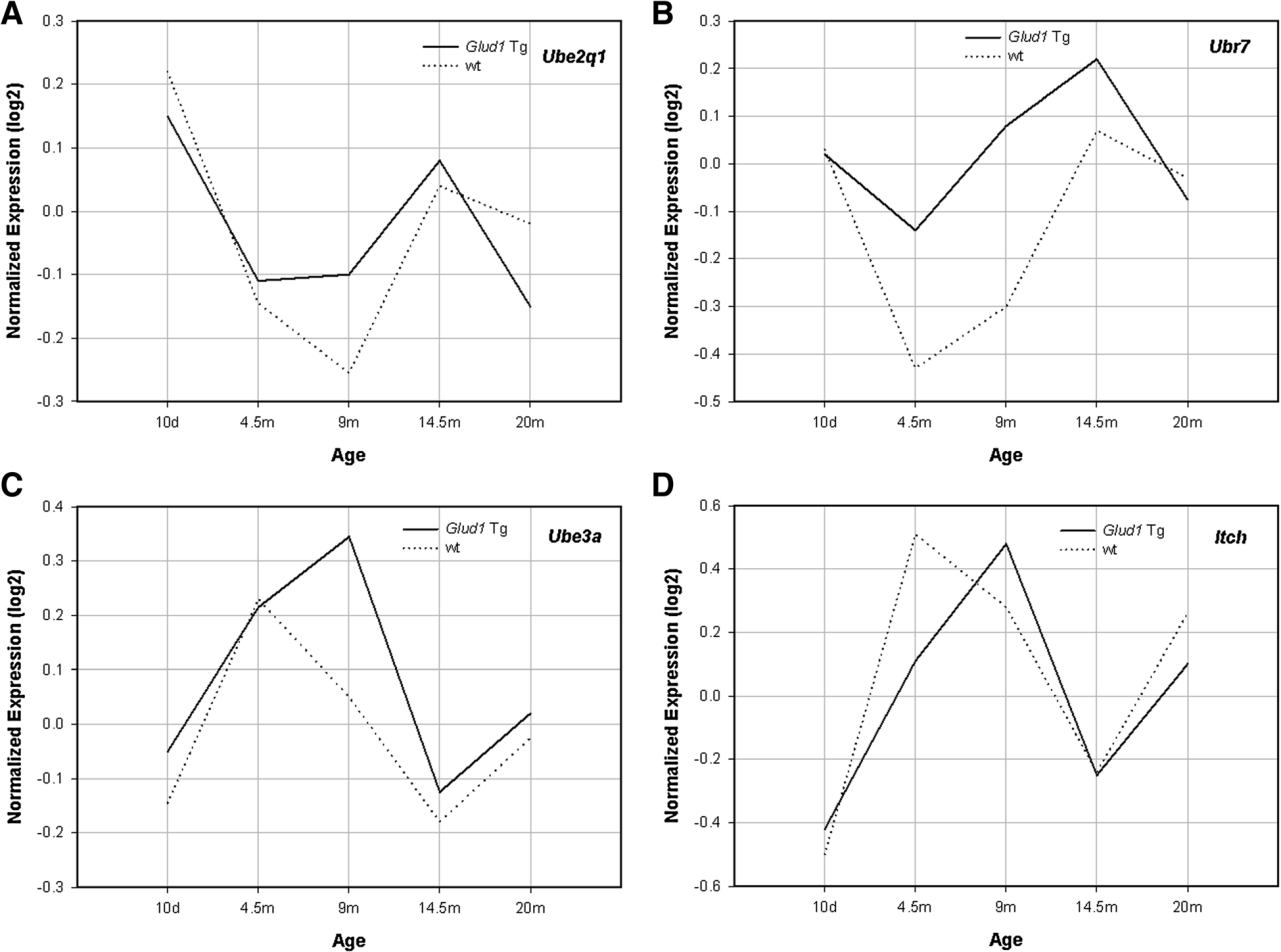
**Patterns of age-related expression of four genes associated with protein ubiquitination.** The genes are Ube2q1 **(A)**, Ubr7 **(B)**, Ube3a **(C)**, and Itch **(D)**. Shown on the Y-axis are the logarithmic transformations (log2) of RMA-normalized expression values. The lines connecting the points are: solid line, Tg; dotted line, wt.

Increases in protein ubiquitination and degradation are a characteristic of cellular responses to stress, occur in the CNS during aging and in age-associated neurodegenerative diseases, and are collectively referred to as altered proteostasis [71,72]. In the present study, a number of protein ubiquitination-related genes showed significant differential expression between the *Glud1* and wt mouse hippocampus, with the Tg hippocampus exhibiting higher levels of expression than the wt across several age groups (Figure 6). Among the genes related to protein ubiquitination were two E2 ubiquitin conjugating enzymes, *Ube2q1* (ubiquitin-conjugating enzyme E2q) and *Hip2* (or *Ube2k*), huntingtin interacting protein 2 (or ubiquitin conjugating enzyme E2k). *Ube2q1* is associated with the endocytic pathway and the proteasomal degradation of proteins [73], while *Hip2* is involved in ubiquitination and aggregation of polyglutamine-containing proteins such as the protein huntingtin in Huntington’s disease [74]. In addition to the two E2 ubiquitin-conjugating enzymes, three genes, *Ubr7*, *Ube3a* and *Itch*, coding for E3 ubiquitin-protein ligases were also differentially expressed in Tg *vs.* wt mice, especially at the age of 9 months (Figure 6). In terms of the role of these genes in CNS function, *Ubr7* is one of fifty genes whose mutations are linked to autosomal recessive intellectual disabilities [75], while suppression of the expression of *Ube3a* in the hippocampus and cerebellum of children leads to the clinical syndrome known as Angelman’s syndrome characterized by seizures and mental and developmental disabilities [76]. *Itch* codes for an E3 ligase that is associated with endothelin A and is part of the endosomal degradation pathway in cells of the nervous system [77].

As pointed out by others [78,79], the expression of these genes may enhance axonal growth, dendritic spine elongation and synapse formation during early development or later in life, thus determining both the structure and function of synapses through ubiquitination and proteasomal degradation of key proteins. Included among the proteins whose placement and cycling at synapses are the Glu receptor proteins in glutamatergic synapses. Such changes in the rates of placement and removal of macromolecules from synapses may alter synaptic transmission and thus have a protective role in chronically stimulated synapses, such as those that exist in the hippocampus of the *Glud1* Tg mice.

Several additional genes related to ubiquitination and protein degradation, were over-expressed in the Tg as compared with the wt hippocampus, including *Cul4a* (ubiquitination complex E3 ligase component), a gene which when over-expressed offers protection against the damage induced by hypoxia and ischemia [80,81]; *Fbxw1b* (F-box and WD-containing 1b) which codes for a component of a ubiquitination complex that includes CUL4 and which leads to proteasomal degradation of targeted proteins [82]; *Rab40c*, a member of an ubiquitin E3 ligase complex and one that is highly expressed in oligodendrocytes and may be involved in myelin formation or repair [83]; and, *Usp7, 19,* and *20* (ubiquitin specific proteases 7, 19, and 20) and *Psmd14* (proteasome 26 S subunit non-ATPase 14), a proteasome component involved in the degradation of proteins. Thus, the expression patterns of the genes for the ubiquitin conjugating enzymes, the ubiquitin ligases, and the ubiquitin-specific proteases enumerated above were suggestive of increased endosomal transport, protein ubiquitination, and proteasomal degradation of ubiquitinated proteins in *Glud1* Tg mouse hippocampus. Such differential expression might lead to altered dynamics at synapses, altered proteostasis, and possibly higher levels of ER stress in the Tg mice. Yet, even such up-regulation of genes of the ubiquitin proteasome system (UPS) may not be sufficient in meeting the increased demands of protein processing needed to maintain normal function in the *Glud1* Tg mice. This might explain why neurons in the hippocampus of *Glud1* Tg mice, especially in the susceptible CA1 region of the hippocampus, exhibit increasing amounts of ubiquitinated protein aggregates after 14 to 16 months of age [32].

It should be noted, however, that not all genes that code for ubiquitinating enzyme proteins were expressed at higher levels in Tg mice throughout development or aging. An example of a gene that was expressed at higher levels in wt mice during early development as well as in the aged mice is *Birc6* (data not shown), a gene that codes for an E2 ubiquitin-conjugating enzyme containing a UBCc domain and a BIR (baculoviral inhibition of apoptosis protein repeat) domain. Pro-apoptotic proteins are ubiquitinated by BIRC6 and subsequently degraded, and that leads to the suppression of apoptosis of cells [84,85]. In the early developmental phase (10 days to 4.5 months), *Birc6* expression was significantly higher in wt than Tg hippocampus. Such a deficiency in BIRC6 in Tg mice might produce a pro-apoptotic effect during development and maturation of the hippocampus and might be correlated with some of the neuronal losses in the hippocampus of *Glud1* mice [32].

### Differential expression of mitochondria-related genes in Tg vs. wt hippocampus

**Figure 7:**
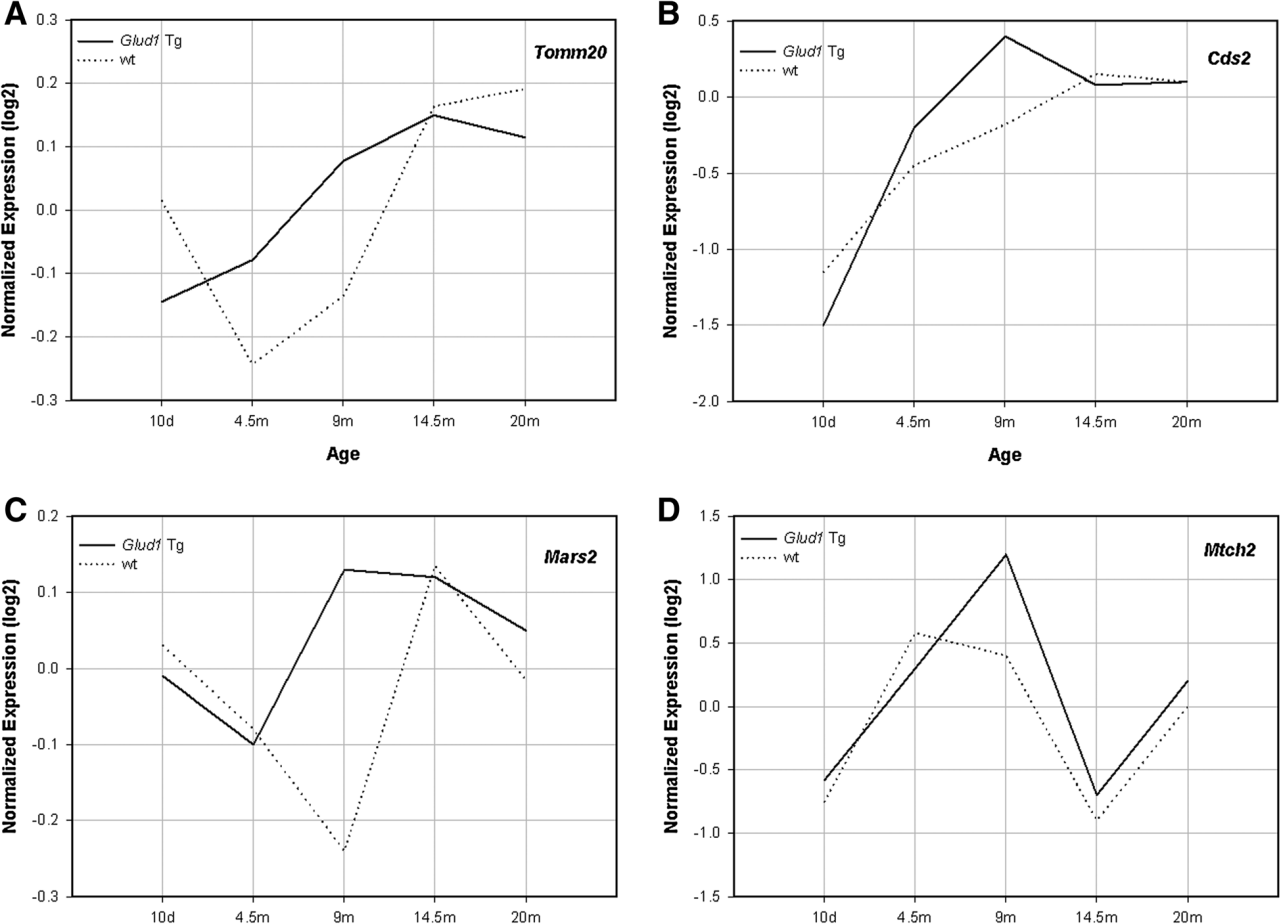
**Comparison of patterns of expression in *Glud1 *Tg vs wt mice of four genes related to mitochondrial structure and function.** The genes are Tomm20 **(A)**, Cds2 **(B)**, Mars2 **(C)**, and Mtch2 **(D)**. RMA-normalized expression values, after logarithmic transformation (log2), are shown on Y-Axis. The lines connecting the points are: solid line, Tg; dotted line, wt.

As described above, genes related to mitochondrial structure and function were also differentially expressed in *Glud1* Tg *vs.* wt mouse hippocampi. Mitochondria play a crucial role in energy metabolism, oxidative stress, apoptosis, calcium regulation, and aging-related neuronal injury. The up-regulation of expression of some of the genes related to mitochondrial function in the Tg mouse hippocampus in young adult to middle aged periods, might be an indication of cellular attempts to sustain normal metabolic activity in the face of increased demand for bioenergetic output. *Tomm20*, *Cds2*, *Mars2*, and *Mtch2* were among the mitochondrial genes identified as exhibiting distinct patterns of differential expression across ages in Tg *vs.* wt mouse hippocampi, especially between the ages of 4.5 and 14.5 months (Figure 7). The expression of*Tomm20* increased steadily from 10 days to 14.5 months of age in Tg mice, whereas the pattern of expression in wt mouse hippocampus exhibited both peaks and valleys over the same period of maturation. The expression of *Cds2* and *Mars2* peaked in *Glud1* mouse hippocampi at 9 months of age and plateaued or moderately decreased after that (Figure 7). The expression of the same two genes in the wt mouse hippocampus lagged behind that in the Tg. The importance of *Cds2* in mitochondrial and brain function was described in a preceding section. The gene *Mars2* codes for a methionyl-tRNA synthetase, a mitochondrial matrix enzyme whose malfunction causes a neurodegenerative phenotype in flies and a recessive form of ataxia in humans [86].

The last in this group of mitochondria-related genes that were differentially expressed in Tg *vs.* wt mouse hippocampi was *Mtch2* (mitochondrial carrier homolog 2) which exhibited a different pattern of expression in both Tg and wt hippocampi than those of *Tomm20*, *Cds2*, and *Mars2* but the overall levels of expression of *Mtch2* were, again, higher in Tg than wt mice (Figure 7). The protein encoded by this gene is an inner mitochondrial membrane protein presumed to function as a transport carrier and to cause mitochondrial membrane depolarization and activation of the cascade of events that lead to mitochondria-associated apoptosis [87]. Although the function of *Mtch2* has been described for liver mitochondria, *Mtch2* is also highly expressed in the mouse hippocampus [88]. Based on the pro-apoptotic function of *Mtch2*, the chronically higher expression of this gene in adult and older Tg mice compared with wt mice, coupled with the lower expression level in Tg than wt mouse hippocampi of the anti-apoptotic gene *Birc6* (described above), might enhance the probability of age-associated neuronal cell losses in the hippocampus of the *Glud1* mice.

### PCR Confirmation of patterns of expression of genes in Tg and wt hippocampus

**Figure 8:**
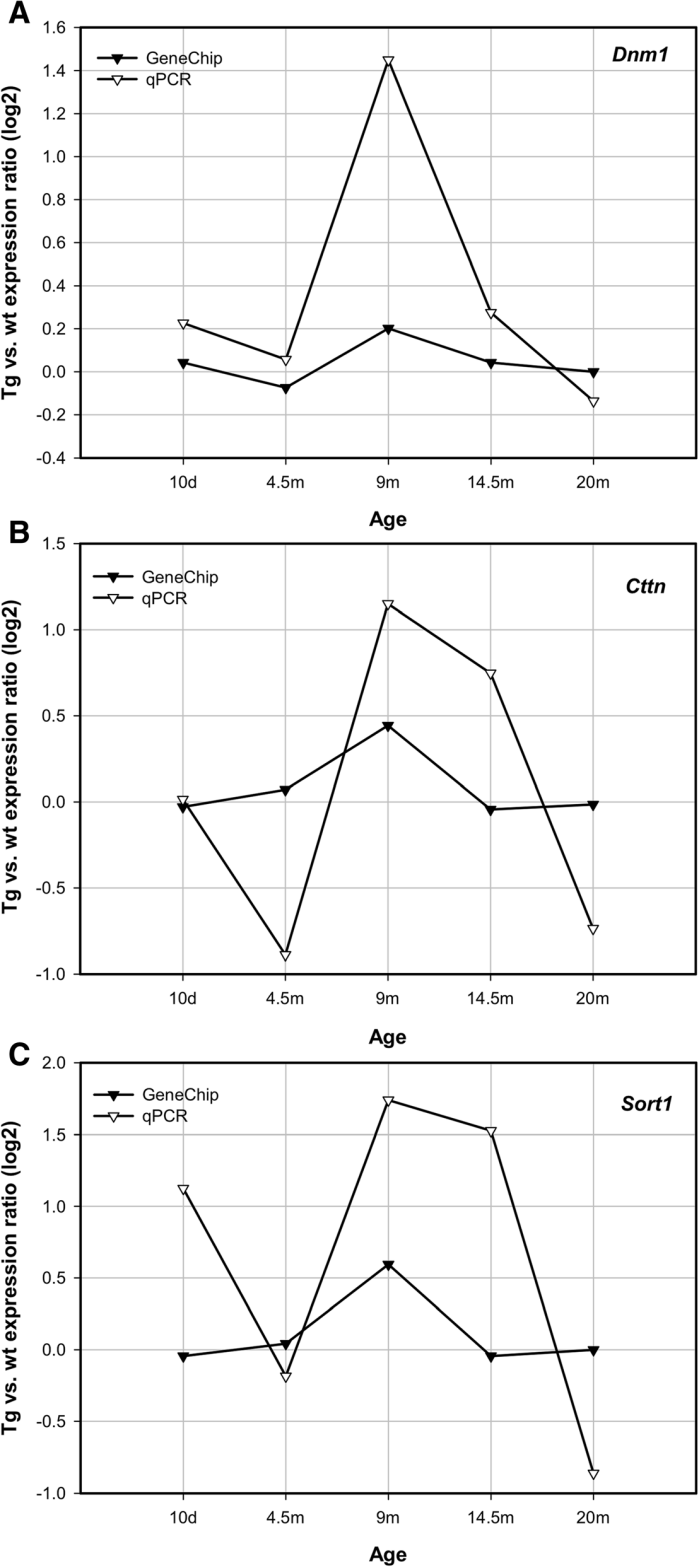
**Comparison of the ratio of Tg/wt expression across age of three significant genes as assessed by GeneChip and qPCR methods.** The genes are Dnm1 **(A)**, Cttn **(B)**, and Sort1 **(C)**. Shown on the Y-axis are the ratios Tg/wt of the logarithmic transformations (log2) of RMA-normalized expression values.

The expression in mouse brain, and especially in the hippocampus, of many of the genes described above was confirmed through reports in the scientific literature and through searches in the Allen Brain Atlas (http://www.brain-map.org/). For most of the genes described in this paper, such confirmation was obtained. In order to confirm a few of the gene expression patterns that we observed using the GeneChips, we performed parallel quantitative PCR analyses of select genes across the age spectrum. The results of the combined PCR and microarray analyses are shown for three such genes in Figure 8. Although the magnitude of differences between Tg and wt hippocampi in different age groups detected by PCR did not exactly match the values obtained by microarray analyses, the overall patterns of the PCR-measured gene expression in these limited number of samples appeared to reproduce those observed by microarray analyses.

## Conclusions

A global assessment of the changes in the categories of *Neuronal development, Nerve process extension, excitability, Synapse formation, Intracellular signaling, Metabolic regulation, Ion transport,* and *Protein homeostasis*, was obtained in the present study by analyzing gene expression changes in the hippocampus of *Glud1* Tg and wt mice from 10 days of life to the old age of 20 months. To our knowledge, this is the first study to explore whole genome transcriptomic changes across the life-span of mice and, specifically, the life-span of two genotypes of C57BL/6 mice, the *Glud1* Tg and wt mice. We could not find literature references or brain maps that present the patterns of gene expression in mouse brain, or mouse hippocampus in particular, across the ages from10 days to 20 months. A few gene expression patterns have been traced up to 56 days of mouse post-natal life by the Allen Brain Atlas (http://www.brain-map.org/). However, because of the limited span of ages examined in this brain mapping effort, these studies do not provide sufficient information about changes in gene expression beyond two months of age.

Among the many changes in gene expression levels during development, maturation, and aging that are described in the present study, a few stand out as being important to neuronal development, function, and survival in the hippocampus. With regard to the aging process, and regardless of the genotype of the mice, there was an age-associated significant decrease in the expression of genes related to *Neurogenesis*, *Neuronal migration*, *Neuronal growth and process elongation*, and an increase in genes related to *Neuro-inflammation*, *Voltage*-*gated channel activity*, and *Regulation of synaptic transmission*. As pointed out in other sections of this paper and in previous publications [35,89], the up-regulation of inflammatory response genes appears to be a common characteristic of aging in the CNS. The up-regulation of voltage-gated channels, primarily potassium channels, is a novel observation. Changes in ion channel expression would be an important component in the control of neuronal function and viability. Increased neuronal excitation due to Glu hyperactivity is known to lead to intracellular and intra-mitochondrial calcium accumulation and, potentially, cell death [2], thus decreases in the excitability that results from increased expression of certain potassium channels would protect neurons from Glu-induced hyper-excitability.

Among the major differences between the *Glud1* Tg and wt mice during development and aging was the differential expression of genes related to *Protein homeostasis*—*i.e.*, genes associated with protein ubiquitination and proteasomal degradation. As was pointed out in other sections in this paper, increases in protein ubiquitination, protein endocytosis, and protein degradation may influence synapse formation and synaptic function during development, and the same processes may affect neuronal excitability and synaptic activity in the CNS during aging. Increased protein ubiquitination and degradation are also a characteristic of cellular stress response and the increased expression of genes related to ubiquitin conjugation during the aging process was previously described for hippocampus nerve cells [90]. In our studies, we observed not only up-regulation of genes for ubiquitin conjugation, but also genes for protein degradation, and, interestingly, these increases in gene expression during the aging process were not of the same magnitude in Tg as in wt mice. These transcriptomic observations indicate a higher stress level, accelerated aging, and possibly increased attempts to control synaptic function and excitability in the *Glud1* Tg mice as compared with wt mice.

The regulation of mitochondrial function across age as determined by gene changes that alter mitochondrial lipid synthesis, cytochrome c oxidation, and protein or solute transport, might be some of the most important aspects of the aging process revealed by the results of our studies. Several studies have previously identified changes in gene expression with advancing age as fitting within the category of mitochondrial dysfunction and oxidative stress [69,91,92]. And as has been pointed out before, altered mitochondrial oxidative phosphorylation and the generation of reactive oxygen species represent the type of dysfunction that leads to cell and organ senescence [93]. Of particular interest in our studies were the differences in genes associated with mitochondrial protein transport, such as TOMM20 and MTCH2, iron transport, enzyme regulation, *e.g.* cytochrome oxidase subunits, and mitochondrial lipid metabolism. We have commented already on the significance of the differential expression of some of these genes in wt *vs.* Tg mouse hippocampi. It is worth pointing out again the relevance in terms of mitochondrial function of the increased expression of TOMM20 in the Tg mice. TOMM20 is an important component of the pathway that maintains normal mitochondrial function and whose loss of function could contribute to the accumulation of dysfunctional mitochondria [94]. The increased expression of TOMM 20 in the *Glud1* Tg mouse hippocampus during development and adulthood may be a direct response to the increased need for GLUD1 protein translocation from the cytoplasm into the mitochondrial matrix. Yet, when one considers the increases in *Tomm20* expression in combination with the other differential changes in mitochondrial gene expression in *Glud1* Tg *vs.* wt mice, it would appear that these differential expression characteristics reflect adaptive or compensatory changes made by brain cells to overcome the increased stress exerted by altered metabolic states in the *Glud1* Tg mice.

## Methods

### Experimental animals

The procedure for generating the Tg mice was described in detail previously [32]. Age-matched wt mice of the same genetic background (C57BL/6) were used as the controls. All animals were housed in a 12 hour light/dark cycle with food and water *ad libitum*. All animal procedures were performed in accordance with guidelines established by the University of Kansas IACUC. Male *Glud1* Tg and wt mice at five different ages were used: 10 day old, 4.5 month, 9 month, 14.5 month, and 20 month old. A total of 3 Tg and 3 wt mice were used for transcriptomic studies of each age group.

### RNA extraction and microarray data generation

The mice used for the transcriptomic studies were anesthetized under CO_2_ anesthesia, the hippocampi dissected out from the brains, flash frozen in liquid nitrogen, and stored at -80°C prior to total RNA extraction. Total RNA samples were extracted from the dissected hippocampi using the Qiagen RNeasy Mini Kit (Qiagen, Valencia, CA, USA). To prepare targets for subsequent GeneChip hybridization, One-Cycle cDNA Synthesis Kit from Affymetrix (Santa Clara, CA, USA) was used according to the manufacturer’s instructions. The Affymetrix GeneChip Mouse Genome 430 2.0 arrays designed to interrogate expression of over 39,000 mouse gene transcripts, were used for the hybridization. Subsequent washing and staining steps were performed on a GeneChip Fluidics 450 Station and the chips were scanned on a GeneChip Scanner 3000 (Affymetrix). Instrument control and data collection were carried out using GeneChip Operating Software (GCOS, ver 1.1.1). In order to minimize experimental variability, all steps in tissue dissection, RNA isolation and microarray operation were performed by a single investigator. The quality and quantity of the original RNA samples and of the cRNA probes generated for array hybridization were determined with an Agilent 2100 Bioanalyzer (Agilent Technologies, Palo Alto, CA, USA), and a NanoDrop ND-1000 Spectrophotometer (Thermo Scientific, Wilmington, DE, USA). The microarray data generated from all chips met the quality control criteria set by Affymetrix, including low background and noise, positive detection of QC probesets such as bioB, percentage of genes called present in normal range (generally between 40-60%), similar scaling factors across all chips, and Acceptable 3’/5’ ratios. All microarray data were deposited into the NCBI GEO (Gene Expression Omnibus) database with accession number GSE48911.

### Transcriptomic data analysis

The GeneChip transcriptomic data collected across the five age points of Tg and wt mouse hippocampus were analyzed: a) at each individual age; b) across the developmental stage (the first three age groups); c) across the aging process (the last three age groups); and d) across all five age points. For each analysis, the GeneChip CEL data were first normalized by the Robust Multiarray Average (RMA) algorithm [95]. Prior to each identification of genes showing significant differential expression patterns between Tg and wt, probesets that were detected to be non-expressed, *i.e.*, those with absence calls in all analyzed samples, were filtered out and excluded from further analyses. In addition, probesets that were designed solely for the purpose of chip quality control (monitoring hybridization target preparation and array hybridization), *i.e.*, those with the ID prefix AFFX, were also filtered out. These filtering steps were employed in order to reduce the number of comparisons in subsequent analyses and thus reduce the FDR.

To identify differentially expressed genes at each individual age point, SAM (Significance Analysis of Microarrays), a supervised learning software for genomic expression data mining [96], was first used with the threshold for calling differential gene expression set at fold change ≥ 1.3 and FDR ≤ 1%. This was followed by Cyber-T analysis [97] in order to confirm expression patterns of differential genes identified by SAM, using the criterion of Bayesian *P* value ≤ 0.05. To identify genes showing both age-dependent change and differential expression between Tg and wt, a software package called EDGE (http://www.genomine.org/edge/) was used [98]. Gene hierarchical clustering was performed using the software GeneSpring GX (Agilent Technologies). Gene Ontology (GO) and Biological Pathway analyses were conducted with DAVID (http://david.abcc.ncifcrf.gov/) [99].

## Competing interests

The authors declare that they have no competing interests.

## Authors’ contributions

XW and EKM conceived of the study and drafted the manuscript. XW also carried out the RNA extraction, GeneChip data generation, bioinformatics data analyses, and participated in real-time quantitative PCR confirmation of the GeneChip data. EKM was also involved in the interpretation of the transcriptomic data and coordinated the study. NDP, MMH, MSS and AAA conducted the real-time quantitative PCR analysis. DH maintained the *Glud1* Tg and wt mice. RP carried out characterization of the animals. All authors read and approved the final manuscript.
